# Racemic mefloquinium chloro­difluoro­acetate: crystal structure and Hirshfeld surface analysis

**DOI:** 10.1107/S2056989018007703

**Published:** 2018-06-05

**Authors:** James L. Wardell, Solange M. S. V. Wardell, Mukesh M. Jotani, Edward R. T. Tiekink

**Affiliations:** a Fundaçaö Oswaldo Cruz, Instituto de Tecnologia em Fármacos-Far Manguinhos, 21041-250 Rio de Janeiro, RJ, Brazil; b CHEMSOL, 1 Harcourt Road, Aberdeen AB15 5NY, Scotland; c Department of Physics, Bhavan’s Sheth R. A. College of Science, Ahmedabad, Gujarat 380001, India; d Research Centre for Crystalline Materials, School of Science and Technology, Sunway University, 47500 Bandar Sunway, Selangor Darul Ehsan, Malaysia

**Keywords:** crystal structure, Mefloquine, salt, hydrogen bonding, Hirshfeld surface analysis

## Abstract

In the racemic title mol­ecular salt, C_17_H_17_F_6_N_2_O^+^·C_2_ClF_2_O_3_^−^ (systematic name: 2-{[2,8-bis­(tri­fluoro­meth­yl)quinolin-4-yl](hy­droxy)meth­yl}piperidin-1-ium chloro­difluoro­acetate), the cation, which is protonated at the piperidine N atom, has the shape of the letter, *L*, with the piperidin-1-ium group being approximately orthogonal to the quinolinyl residue [the C_q_—C_m_—C_m_–N_a_ (q = quinolinyl; m = methine; a = ammonium) torsion angle is 177.79 (18)°]. An intra­molecular, charge-assisted ammonium-*N*—*H*⋯*O*(hydrox­yl) hydrogen bond ensures the hy­droxy-O and ammonium-N atoms lie to the same side of the mol­ecule [O_h_—C_m_—C_m_—N_a_ (h = hydrox­yl) = −59.7 (2)°]. In the crystal, charge-assisted hydroxyl-O—H⋯O^−^(carboxyl­ate) and ammonium-N^+^—H⋯O^−^(carboxyl­ate) hydrogen bonds generate a supra­molecular chain along [010]; the chain is consolidated by C—H⋯O inter­actions. Links between chains to form supra­molecular layers are of the type C—Cl⋯π(quinolinyl-C_6_) and the layers thus formed stack along the *a*-axis direction without directional inter­actions between them. The analysis of the calculated Hirshfeld surface points to the dominance of F⋯H contacts to the surface (40.8%) with significant contributions from F⋯F (10.5%) and C⋯F (7.0%) contacts.

## Abstract 2

The l-shaped cation in the title salt arises from a nearly orthogonal disposition of the piperidin-1-ium ring with respect to the piperidin-1-ium group. Supra­molecular chains arise in the crystal as a result of O—H⋯O and N—H⋯O hydrogen bonding.

## Chemical context   

Practical inter­est in compounds related to the title salt relates to the biological activity of Mefloquine ([2,8-bis­(tri­fluoro­meth­yl)quinolin-4-yl]-piperidin-2-yl­methanol). This arises when the racemic compound is reacted with HCl: the resulting salt, [(R*,S*)-(2-{[2,8-bis­(tri­fluoro­meth­yl)quinolin-4-yl](hy­droxy­meth­yl)piperidin-1-ium chloride is an anti-malarial drug, being effective against the causative agent, *Plasmodium falciparum* (Maguire *et al.*, 2006 ▸). Subsequently, diverse pharmaceutical potential has been disclosed, namely, as anti-bacterial (Mao *et al.*, 2007 ▸), anti-mycobacterial (Gonçalves *et al.*, 2012 ▸) and as anti-cancer agents (Rodrigues *et al.*, 2014 ▸). With the preceding facts in mind, it is not surprising that crystallography has played a key role in establishing the mol­ecular structures of this class of compound. Of particular crystallographic inter­est has been the characterization of a pair of kryptoracemates of mefloquinium salts in recent years (Jotani *et al.*, 2016 ▸; Wardell, Wardell *et al.*, 2016 ▸). The phenomenon of kryptoracemic behaviour has been reviewed in the last decade for both organic and coordination compounds (Fábián & Brock, 2010 ▸; Bernal & Watkins, 2015 ▸). Briefly, for a material to be classified as kryptoracemic, it must satisfy the following crystallographic criteria: the space group must be one of the 65 Sohncke space groups, *i.e*. lacking an inversion centre, rotatory inversion axis, glide plane or a mirror plane, and *Z*′ would usually be greater than 1 (unless the mol­ecule lies on a rotation axis). In a continuation of structural studies of Mefloquine derivatives (Wardell *et al.*, 2011 ▸; Wardell, Jotani *et al.*, 2016 ▸), herein the crystal and mol­ecular structures of the title salt, (I), isolated from the 1:1 crystallization of racemic Mefloquine and chloro­difluoro­acetic acid are described along with an analysis of its calculated Hirshfeld surface.
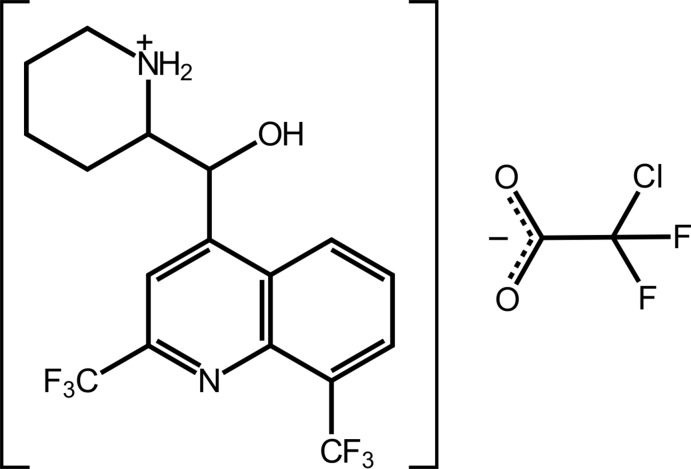


## Structural commentary   

**Figure 1:**
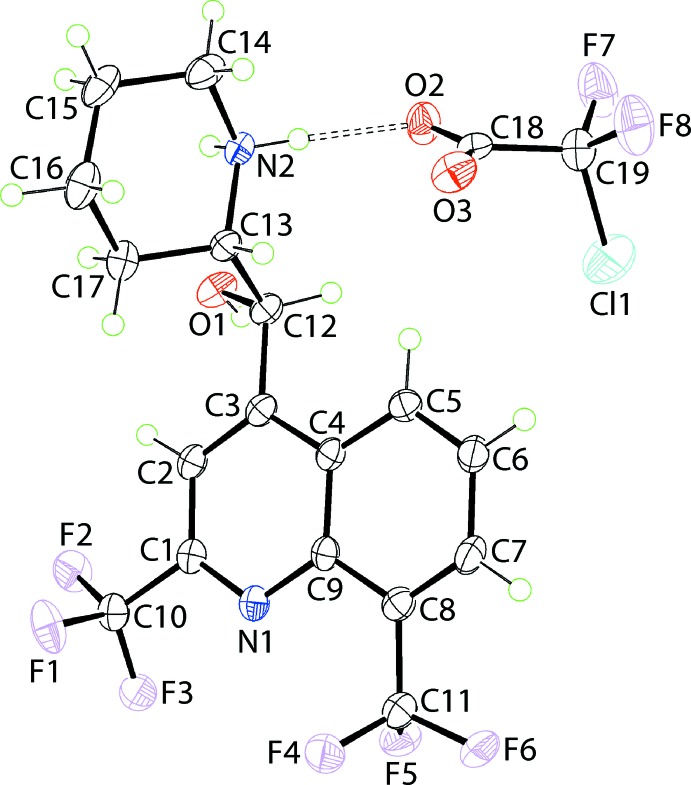
The mol­ecular structures of the ions comprising the asymmetric unit of (I) showing the atom-labelling scheme and displacement ellipsoids at the 70% probability level. The dashed line signifies the N—H⋯O hydrogen bond.

The ions comprising the asymmetric unit of (I) are shown in Fig. 1 ▸. The illustrated cation has two chiral centres, namely *R* at C12 and *S* at C13, *i.e*. it is the [(+)-erythro-mefloquinium] isomer. However, it should be noted that the centrosymmetric unit cell has equal numbers of the other *S*-,*R*- enanti­omer, indicating that no resolution occurred during the crystallization experiment as has been observed in some of the earlier studies (see *Chemical context*). The pattern of hydrogen-bonding inter­actions involving the ammonium-N—H H atoms (see *Supra­molecular features*) provides confirmation of protonation at the N2 atom during crystallization and, therefore, the formation of a piperidin-1-ium cation. At the same time, delocalization of the π-electron density over the carboxyl­ate residue is confirmed by the equivalence of the C18—O2, O3 bond lengths, *i.e*. 2 × 1.238 (3) Å.

The quinolinyl residue is not strictly planar with the r.m.s. deviation for the ten fitted non-H atoms being 0.0399 Å. This is also reflected in the dihedral angle formed between the (N1,C1–C4,C9) and (C4–C9) rings of 3.95 (15) Å. This aspect of the structure notwithstanding, the hydroxyl-O and ammonium-N atoms lie to opposite sides of the plane through the quinolinyl residue. This is seen in the value of the C2—C3—C12—O1 torsion angle of −20.3 (3)° *cf*. with that of 177.79 (18)° for C3—C12—C13—N2. The latter angle indicates the piperidin-1-ium residue is almost perpendicular to the quinolinyl residue with the methyl­ene-C17 group orientated towards the fused-ring system as seen in the gauche C3—C12—C13—C17 torsion angle of −60.7 (3)°. The observed conformation, whereby the hy­droxy-O and ammonium-N atoms lie to the same side of the mol­ecule [the O1—C12—C13—N2 torsion angle is −59.7 (2)°], is stabilized by an intra­molecular, charge-assisted ammonium-N2^+^—H⋯O1(hydrox­yl) hydrogen bond, Table 1 ▸. In general terms, the shape of the cation is based on the letter, *L*.

The anion in (I) adopts a conformation where the Cl1 atom lies to one side of the O_2_C_2_ plane [r.m.s. deviation = 0.0089 Å], with the O2—C18—C19—Cl1 torsion angle being −93.3 (2)°, and the F7 and F8 atoms lying to the other side, the O2—C18—C19—F7, F8 torsion angles = 28.8 (3) and 146.3 (2)°, respectively. The conformation of the CClF_2_ residue in (I) has been observed in the structure of the acid (Schilling & Mootz, 1995 ▸), the acid monohydrate and tetra­hydrate (Dahlems *et al.*, 1996 ▸) and in salts, *e.g*. with mono-protonated 1,4-di­aza­bicyclo­[2.2.2]octane (dabco), *i.e*. 4-aza-1-azoniabi­cyclo­[2.2.2]octane, for which three independent ion pairs comprise the asymmetric unit (Shi *et al.*, 2013 ▸).

## Supra­molecular features   

**Figure 2:**
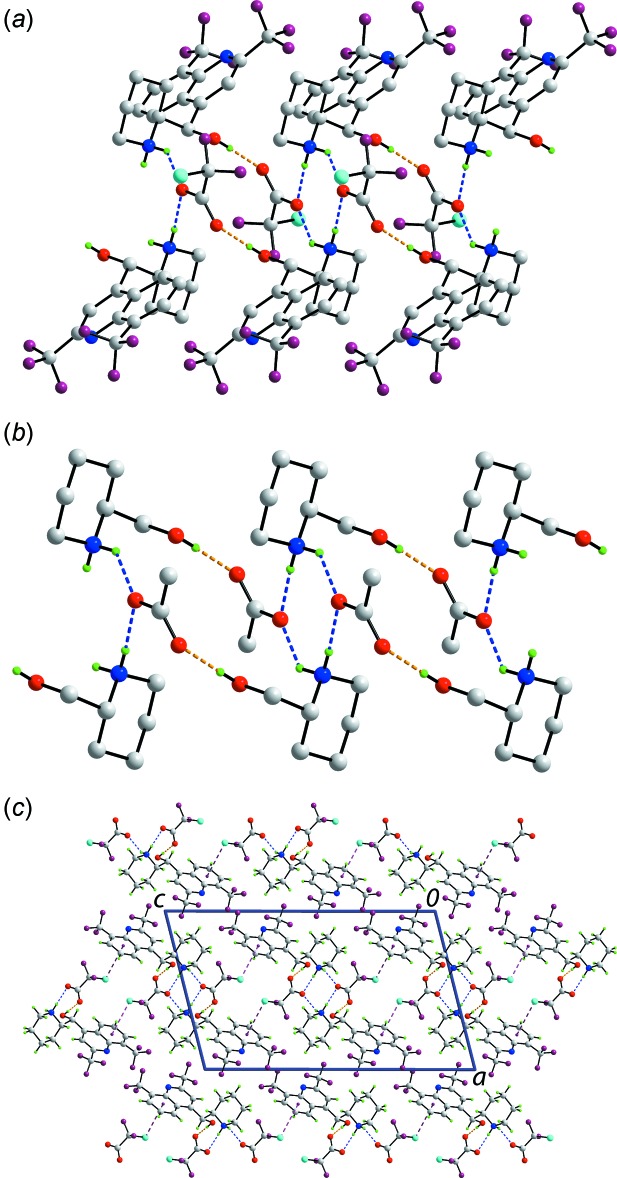
Mol­ecular packing in (I): (*a*) The supra­molecular chain along the *b*-axis direction, being sustained by O—H⋯O and N—H⋯O hydrogen bonding with non-participating H atoms omitted, (*b*) a simplified view of the chain highlighting the formation of the eight- and 18-membered supra­molecular synthons and (*c*) a view of the unit-cell contents shown in projection down the *b*-axis direction. The O—H⋯O, N—H⋯O and Cl⋯π inter­actions are shown as orange, blue and purple dashed lines, respectively.

The presence of charge-assisted hydroxyl-O—H⋯O^−^(carb­oxyl­ate) and ammonium-N^+^—H⋯O^−^(carboxyl­ate) hydrogen bonding features prominently in the mol­ecular packing of (I) and leads to a supra­molecular chain propagating along the *b-*axis direction, Fig. 1 ▸ *a* and Table 1 ▸. The ammonium-N^+^—H⋯O^−^(carboxyl­ate) hydrogen bonds link two cations and two anions about a centre of inversion to form eight-membered {⋯HNH⋯O}_2_ synthons, Fig. 2 ▸ *b*. These are linked into a supra­molecular chain *via* hydroxyl-O—H⋯O^−^(carboxyl­ate) hydrogen bonding, which leads to 18-membered {⋯OCO⋯HNC_2_OH}_2_ synthons, Fig. 2 ▸ *b*. In this scheme, the carboxyl­ate-O2 atom forms two hydrogen bonds. Additional stability to the supra­molecular chain is afforded by quinolinyl-C—H⋯O(carboxyl­ate) and methyl­ene-C—H⋯O(hydrox­yl) inter­actions, Table 1 ▸. The chains are connected into layers *via* C—Cl⋯π(C4–C9) inter­actions, Table 1 ▸. The layers stack along the *a*-axis direction without directional inter­actions between them, Fig. 2 ▸ *c*.

## Hirshfeld surface analysis   

**Figure 3:**
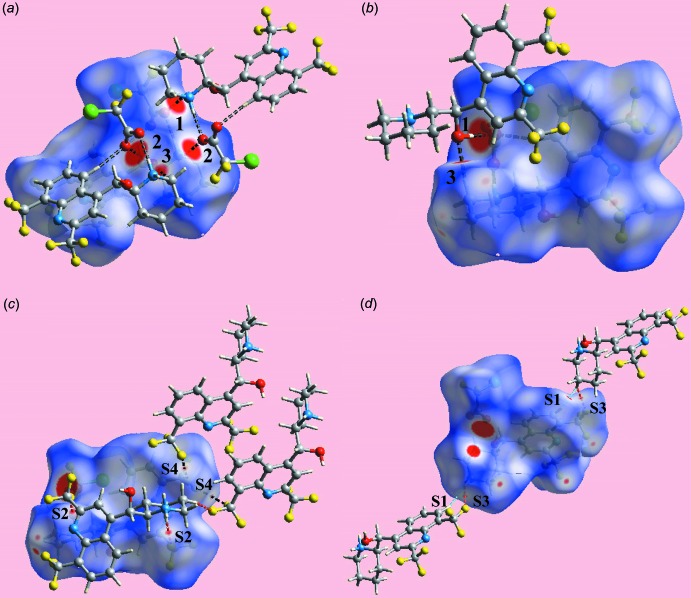
Views of the Hirshfeld surface of (I) mapped over *d*_norm_ in the range −0.077 to +1.575 au, highlighting: (*a*) and (*b*) inter­molecular hydrogen bonds (with labels 1–3) by black-dashed lines, and (*c*) and (*d*) short inter­atomic H⋯H, F⋯H and F⋯F contacts (with labels S1–S4) by sky-blue, red and black dashed lines, respectively.

**Figure 4:**
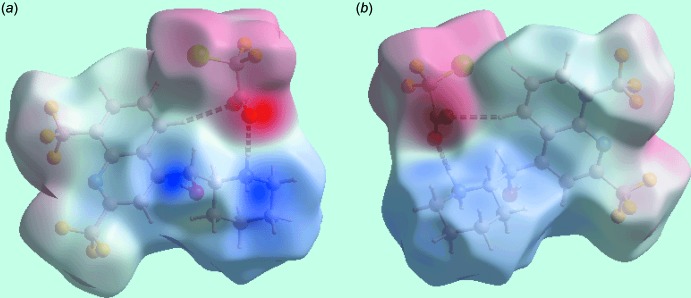
Two views of the Hirshfeld surface of (I) mapped over the electrostatic potential in the range −0.133 to + 0.219 au. The red and blue regions represent negative and positive electrostatic potentials, respectively.

**Figure 5:**
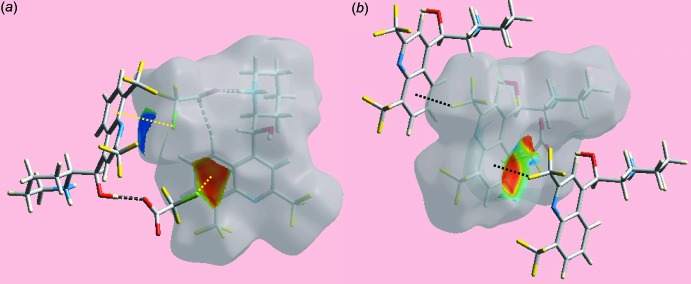
Two views of Hirshfeld surface of (I) mapped over the shape-index property highlighting (*a*) C—Cl⋯π and (*b*) C—F⋯π contacts by yellow and black dotted lines, respectively

The Hirshfeld surface calculations for the title salt (I) were performed in accord with an earlier publication on a related salt (Jotani *et al.*, 2016 ▸) and satisfactorily describe the additional influence of inter­atomic halogen–halogen, halogen–hydrogen and halogen⋯π contacts upon the packing. In addition to bright-red spots on the Hirshfeld surfaces mapped over *d*_norm_ in Fig. 3 ▸ *a* and *b* (labelled 1–3), corresponding to inter­molecular O—H⋯O, N—H⋯O and C—H⋯O inter­actions, Table 1 ▸, the presence of tiny faint-red spots, having labels S1–S4 in Fig. 3 ▸ *c* and *d*, indicate the influence of short inter­atomic H⋯H, F⋯H/H⋯F and F⋯F contacts [Table 2 ▸; calculated in *CrystalExplorer3.1* (Wolff *et al.*, 2012 ▸)]. On the Hirshfeld surfaces mapped over electrostatic potential in Fig. 4 ▸, the donors and acceptors of inter­molecular hydrogen bonds are illustrated through the appearance of blue and red regions corresponding to positive and negative electrostatic potential, respectively. The presence of inter­molecular side-on C—halogen⋯π inter­actions namely C19—Cl1⋯π(C4–C9) and C10—F3⋯π(C4–C9), Table 1 ▸, are evident from the Hirshfeld surfaces mapped with shape-index property illustrated in Fig. 5 ▸.

**Figure 6:**
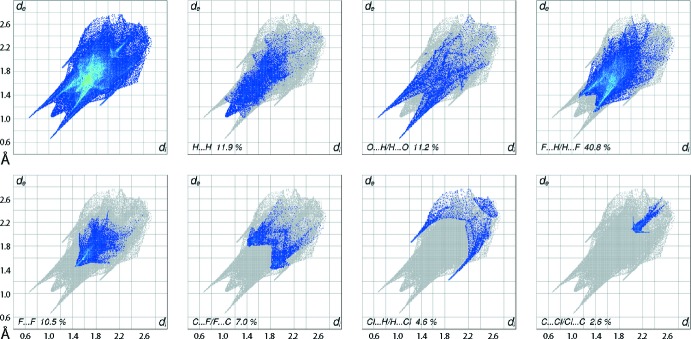
The full two-dimensional fingerprint plot for (I) and those delineated into H⋯H, O⋯H/H⋯O, F⋯H/H⋯F, F⋯F, C⋯F/F⋯C, Cl⋯H/H⋯Cl and C⋯Cl/Cl⋯C contacts.

The overall two-dimensional fingerprint plot and those delineated (McKinnon *et al.*, 2007 ▸) into H⋯H, O⋯H/H⋯O, F⋯H/H⋯F, F⋯F, C⋯F/F⋯C, Cl⋯H/H⋯Cl and C⋯Cl/Cl⋯C contacts are illustrated in Fig. 6 ▸; the percentage contributions from the different inter­atomic contacts to the Hirshfeld surface are summarized in Table 3 ▸. The formation of a salt between the piperidinium cation and carboxyl­ate anion through the charge-assisted hydrogen bonds and the presence of a number of H⋯Cl, F and O contacts result in the relatively small, *i.e*. 11.9%, contribution from H⋯H contacts to the Hirshfeld surface. Conversely, the relative high number of fluorine atoms lying on the surfaces of both the cation and anion, largely participating in F⋯H contacts, gives rise to their providing the greatest contribution, *i.e*. 40.8%, to the surface.

In the fingerprint plot delineated into H⋯H contacts in Fig. 6 ▸, the short inter­atomic H⋯H contact involving quinoline-H7 and methyl­ene-H15*B*, both derived from the cation, Table 2 ▸, is viewed as pencil-like tip at *d*_e_ + *d*_i_ ∼2.0 Å. In the fingerprint plot delineated into O⋯H/H⋯O contacts, the spikes associated with the N—H⋯O hydrogen bonds and C—H⋯O inter­actions are merged within the plot. The obvious feature in the plot is a pair of spikes with tips at *d*_e_ + *d*_i_ ∼1.8 Å, which correspond to the most dominant O—H⋯O hydrogen bond; this is also responsible for most of the points concentrated in the narrower region of spikes. The influence of short inter­atomic halogen–hydrogen and halogen–halogen contacts in the crystal, Table 2 ▸, is observed as a pair of forceps-like tips at *d*_e_ + *d*_i_ ∼2.5 Å (F⋯H) and 3.0 Å (Cl⋯H), and an arrow-shaped tip at *d*_e_ + *d*_i_ ∼2.8 Å in the fingerprint plots delineated into F⋯H/H⋯F, Cl⋯H/H⋯Cl and F⋯F contacts, respectively. The involvement of chloride and fluoride atoms in C-halogen⋯π contacts, Table 1 ▸, results in the small but significant percentage contribution from C⋯F/F⋯C and C⋯Cl/Cl⋯C contacts to the Hirshfeld surface, Table 3 ▸. These inter­molecular contacts are also characterized as forceps-like and anchor-shaped distributions of points in the fingerprint plots delineated into the respective contacts, Fig. 6 ▸. The small percentage contribution from other remaining inter­atomic contacts summarized in Table 3 ▸ have negligible effect on the packing in the crystal.

## Database survey   

Kryptoracemic behaviour is rare and is found in only 0.1% of all organic structures (Fábián & Brock, 2010 ▸). This observation clearly implies that 99.9% of racemic compounds, mol­ecules with meso symmetry and achiral mol­ecules will crystallize about a centre of inversion. Given there are fewer than 30 structures containing Mefloquine/derivatives of Mefloquine included in the Cambridge Structural Database (Groom *et al.*, 2016 ▸), the reporting of two kryptoracemates of mefloquinium cations in recent times (Jotani *et al.*, 2016 ▸; Wardell, Wardell *et al.*, 2016 ▸) suggests a higher than anti­cipated propensity for this phenomenon. The two examples were isolated from attempts at chiral resolution of Mefloquine with carb­oxy­lic acids. In the first of the two reported structures, the asymmetric unit comprised a pair of pseudo-enanti­omeric mefloquinium cations with the charge-balance provided by chloride and 4-fluoro­benzene­sulfonate anions (Jotani *et al.*, 2016 ▸). In the second example, again two mefloquinium cations are pseudo-racemic, with the charge-balance provided by two independent 3,3,3-tri­fluoro-2-meth­oxy-2-phenyl­propano­ate anions, *i.e*. (+)-PhC(CF_3_)(OMe)CO_2_^−^ (Wardell, Wardell *et al.*, 2016 ▸). The appearance of kryptoracemic salts of mefloquinium with non-chiral and chiral counter-ions warrants further investigation into this comparatively rare behaviour in order to reveal the reasons for such crystallization outcomes.

## Synthesis and crystallization   

A solution of mefloquinium chloride (1 mmol) and sodium di­fluoro­choro­acetate (1 mmol) in EtOH (10ml) was refluxed for 20 mins. The reaction mixture was left at room temperature and after two days, colourless crystals of the title salt, (I), were collected; M.p. 473–475 K.^1^H NMR (DMSO-*d*_6_) δ: 1.20–1.35 (2H, *m*), 1.55–1.75 (4H, *m*), 3.04 (1H, *br t*), 3.53 (1H, *br d*), 5.90 (1H, *s*), 6.94 (1H, *br d*), 8.01 (1H, *t*, *J* = 8.0 Hz), 8.13 (1H, *s*), 8.42 (1H, *d*, *J* = 8.02 Hz), 8.72 (1*H*, *d*, *J* = 8.0 Hz), 9.48 (1H, *br s*); N—H H not observed. ^13^C NMR (DMSO-*d*_6_) δ: 21.43 (2×), 21.59, 44.51, 58.90, 67.85, 1135.50. 121.17 (*J*_C,F_ = 273.8 Hz), 121.21 (*J*_C,F_ = 311.0 Hz), 123.64 (*J*_C,F_ = 271.7.8 Hz), 126.37, 127.93 (*J*_C,F_ = 29.2 Hz), 128.32, 128.68. 129.9 (*J*_C,F_ = 5.2 Hz), 142.78, 146.73 (*J*_C,F_ = 34.5 Hz), 150.97, 159.82 (*J*_C,F_ = 25.2 Hz). ^19^F NMR (DMSO-*d*_6_) δ: −58.65, −58.84, −66.68. IR (cm^−1^) 3300–2400 (*s*,*v br*), 1662 (*s*).

## Refinement   

Crystal data, data collection and structure refinement details are summarized in Table 4 ▸. The carbon-bound H atoms were placed in calculated positions (C—H = 0.95–1.00 Å) and were included in the refinement in the riding-model approximation, with *U*_iso_(H) set to 1.2*U*_eq_(C). The O- and N-bound H atoms were refined with the distance restraints O—H = 0.84±0.01 and 0.88±0.01 Å, respectively, and with *U*_iso_(H) = 1.5*U*_eq_(O) and 1.2*U*_eq_(N), respectively.

## Supplementary Material

Crystal structure: contains datablock(s) I, global. DOI: 10.1107/S2056989018007703/hb7752sup1.cif

Structure factors: contains datablock(s) I. DOI: 10.1107/S2056989018007703/hb7752Isup2.hkl

Supporting information file. DOI: 10.1107/S2056989018007703/hb7752Isup3.cml

CCDC reference: 1844854

Additional supporting information:  crystallographic information; 3D view; checkCIF report

## Figures and Tables

**Table 1:** Hydrogen-bond geometry (Å, °) *Cg*1 is the centroid of the (C4–C9) ring.

*D*—H⋯*A*	*D*—H	H⋯*A*	*D*⋯*A*	*D*—H⋯*A*
N2—H2*N*⋯O1	0.89 (2)	2.34 (2)	2.722 (3)	106 (2)
O1—H1*O*⋯O3^i^	0.84 (2)	1.83 (2)	2.668 (3)	178 (3)
N2—H1*N*⋯O2	0.89 (2)	1.92 (2)	2.808 (3)	177 (2)
N2—H2*N*⋯O2^ii^	0.89 (2)	2.05 (2)	2.776 (3)	138 (2)
C5—H5⋯O3	0.95	2.45	3.367 (3)	162
C14—H14*B*⋯O1^iii^	0.99	2.39	3.362 (3)	166
C19—Cl1⋯*Cg*1^iv^	1.74 (1)	3.91 (1)	4.208 (3)	88 (1)
C10—F3⋯*Cg*1^i^	1.33 (1)	3.09 (1)	3.762 (3)	110 (1)

Symmetry codes: (i) 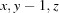; (ii) 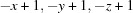; (iii) 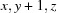; (iv) 
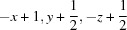
.

**Table 2:** Summary of short inter­atomic contacts (Å) in (I)

Contact	Distance	Symmetry operation
H7⋯H15*B*	2.08	*x*, 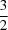 − *y*, − 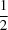 + *z*
F1⋯H16*B*	2.56	2 − *x*, 1 − *y*, 1 − *z*
F6⋯H15*B*	2.58	*x*, 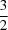 − *y*, − 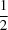 + *z*
F4⋯F5	2.903 (2)	2 − *x*, 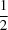 + *y*, 1 − *z*

**Table 3:** Percentage contributions of inter­atomic contacts to the Hirshfeld surface for (I)

	Percentage contribution
Contact	(I)
H⋯H	11.9
F⋯H/H⋯F	40.8
O⋯H/H⋯O	11.2
F⋯F	10.5
C⋯F/F⋯C	7.0
Cl⋯H/H⋯Cl	4.6
C⋯H/H⋯C	3.5
F⋯Cl/Cl⋯F	3.1
C⋯Cl/Cl⋯C	2.6
N⋯H/H⋯N	2.2
C⋯C	0.6
O⋯O	0.3
N⋯F/F⋯N	0.3
C⋯N/N⋯C	0.2
C⋯O/O⋯C	0.1
O⋯Cl/Cl⋯O	0.1

**Table 4:** Experimental details

Crystal data	
Chemical formula	C_17_H_17_F_6_N_2_O^+^·C_2_ClF_2_O_2_^−^
*M*_r_	508.79
Crystal system, space group	Monoclinic, *P*2_1_/*c*
Temperature (K)	120
*a*, *b*, *c* (Å)	14.4535 (4), 6.3387 (2), 23.9040 (8)
β (°)	104.214 (2)
*V* (Å^3^)	2122.95 (12)
*Z*	4
Radiation type	Mo *K*α
μ (mm^−1^)	0.27
Crystal size (mm)	0.62 × 0.20 × 0.06

Data collection	
Diffractometer	Bruker–Nonius Roper CCD camera on κ-goniostat
Absorption correction	Multi-scan (*SADABS*; Sheldrick, 2007 ▸)
*T*_min_, *T*_max_	0.623, 0.746
No. of measured, independent and observed [*I* > 2σ(*I*)] reflections	19411, 4799, 3311
*R*_int_	0.054
(sin θ/λ)_max_ (Å^−1^)	0.649

Refinement	
*R*[*F* ^2^ > 2σ(*F* ^2^)], *wR*(*F* ^2^), *S*	0.052, 0.142, 1.04
No. of reflections	4799
No. of parameters	307
No. of restraints	3
H-atom treatment	H atoms treated by a mixture of independent and constrained refinement
Δρ_max_, Δρ_min_ (e Å^−3^)	0.94, −0.83

Computer programs: *DENZO* (Otwinowski & Minor, 1997 ▸), *COLLECT* (Hooft, 1998 ▸), *SHELXS* (Sheldrick, 2008 ▸), *SHELXL2014/7* (Sheldrick, 2015 ▸), *ORTEP-3 for Windows* (Farrugia, 2012 ▸), *DIAMOND* (Brandenburg, 2006 ▸) and *publCIF* (Westrip, 2010 ▸).

## supplementary crystallographic information

### Crystal data

**Table:**

C_17_H_17_F_6_N_2_O^+^·C_2_ClF_2_O_2_^−^	*F*(000) = 1032
*M**_r_* = 508.79	*D*_x_ = 1.592 Mg m^−^^3^
Monoclinic, *P*2_1_/*c*	Mo *K*α radiation, λ = 0.71073 Å
*a* = 14.4535 (4) Å	Cell parameters from 17332 reflections
*b* = 6.3387 (2) Å	θ = 2.9–27.5°
*c* = 23.9040 (8) Å	µ = 0.27 mm^−^^1^
β = 104.214 (2)°	*T* = 120 K
*V* = 2122.95 (12) Å^3^	Lath, colourless
*Z* = 4	0.62 × 0.20 × 0.06 mm

### Data collection

**Table:**

Bruker–Nonius Roper CCD camera on κ-goniostat diffractometer	4799 independent reflections
Radiation source: Bruker–Nonius FR591 rotating anode	3311 reflections with *I* > 2σ(*I*)
Graphite monochromator	*R*_int_ = 0.054
Detector resolution: 9.091 pixels mm^-1^	θ_max_ = 27.5°, θ_min_ = 3.0°
φ & ω scans	*h* = −18→18
Absorption correction: multi-scan (*SADABS*; Sheldrick, 2007)	*k* = −8→8
*T*_min_ = 0.623, *T*_max_ = 0.746	*l* = −31→30
19411 measured reflections	

### Refinement

**Table:**

Refinement on *F*^2^	3 restraints
Least-squares matrix: full	Hydrogen site location: mixed
*R*[*F*^2^ > 2σ(*F*^2^)] = 0.052	H atoms treated by a mixture of independent and constrained refinement
*wR*(*F*^2^) = 0.142	*w* = 1/[σ^2^(*F*_o_^2^) + (0.0643*P*)^2^ + 1.2072*P*] where *P* = (*F*_o_^2^ + 2*F*_c_^2^)/3
*S* = 1.04	(Δ/σ)_max_ < 0.001
4799 reflections	Δρ_max_ = 0.94 e Å^−^^3^
307 parameters	Δρ_min_ = −0.83 e Å^−^^3^

### Special details

**Table:**

Geometry. All esds (except the esd in the dihedral angle between two l.s. planes) are estimated using the full covariance matrix. The cell esds are taken into account individually in the estimation of esds in distances, angles and torsion angles; correlations between esds in cell parameters are only used when they are defined by crystal symmetry. An approximate (isotropic) treatment of cell esds is used for estimating esds involving l.s. planes.

### Fractional atomic coordinates and isotropic or equivalent isotropic displacement parameters (Å^2^)

**Table:**

	*x*	*y*	*z*	*U*_iso_*/*U*_eq_	
F1	1.03148 (10)	−0.0703 (2)	0.44433 (7)	0.0399 (4)	
F2	0.91566 (11)	−0.2736 (2)	0.44998 (7)	0.0359 (4)	
F3	0.96175 (11)	−0.2572 (2)	0.37136 (6)	0.0366 (4)	
F4	0.98916 (10)	0.2960 (2)	0.26696 (6)	0.0349 (4)	
F5	0.86497 (10)	0.1175 (2)	0.22440 (6)	0.0336 (4)	
F6	0.89009 (11)	0.4242 (2)	0.19252 (6)	0.0375 (4)	
O1	0.66219 (12)	0.1694 (3)	0.47757 (7)	0.0279 (4)	
H1O	0.6352 (19)	0.078 (4)	0.4540 (10)	0.042*	
N1	0.88608 (13)	0.1322 (3)	0.34721 (8)	0.0216 (4)	
N2	0.64265 (14)	0.5664 (3)	0.51657 (8)	0.0218 (4)	
H1N	0.5904 (12)	0.601 (4)	0.4899 (8)	0.026*	
H2N	0.6290 (17)	0.450 (3)	0.5335 (10)	0.026*	
C1	0.87768 (16)	0.0391 (3)	0.39479 (9)	0.0208 (5)	
C2	0.81459 (16)	0.1003 (4)	0.42804 (10)	0.0229 (5)	
H2	0.8107	0.0219	0.4613	0.027*	
C3	0.75848 (16)	0.2756 (4)	0.41176 (9)	0.0208 (5)	
C4	0.76778 (16)	0.3898 (4)	0.36220 (9)	0.0210 (5)	
C5	0.71864 (16)	0.5805 (4)	0.34349 (10)	0.0234 (5)	
H5	0.6760	0.6371	0.3642	0.028*	
C6	0.73220 (17)	0.6835 (4)	0.29583 (10)	0.0264 (5)	
H6	0.7007	0.8140	0.2846	0.032*	
C7	0.79223 (18)	0.5987 (4)	0.26324 (10)	0.0272 (5)	
H7	0.8003	0.6718	0.2301	0.033*	
C8	0.83903 (16)	0.4123 (4)	0.27881 (10)	0.0228 (5)	
C9	0.83122 (15)	0.3071 (4)	0.33029 (9)	0.0197 (5)	
C10	0.94619 (17)	−0.1411 (4)	0.41442 (10)	0.0253 (5)	
C11	0.89662 (18)	0.3125 (4)	0.24146 (10)	0.0275 (5)	
C12	0.68616 (16)	0.3397 (4)	0.44507 (10)	0.0222 (5)	
H12	0.6269	0.3899	0.4171	0.027*	
C13	0.72279 (15)	0.5153 (4)	0.48924 (9)	0.0210 (5)	
H13	0.7367	0.6430	0.4681	0.025*	
C14	0.66441 (19)	0.7397 (4)	0.56031 (10)	0.0296 (6)	
H14A	0.6100	0.7582	0.5782	0.036*	
H14B	0.6738	0.8737	0.5412	0.036*	
C15	0.75362 (19)	0.6874 (4)	0.60653 (10)	0.0323 (6)	
H15A	0.7409	0.5645	0.6291	0.039*	
H15B	0.7705	0.8086	0.6332	0.039*	
C16	0.83715 (19)	0.6373 (4)	0.58047 (11)	0.0322 (6)	
H16A	0.8929	0.5940	0.6115	0.039*	
H16B	0.8550	0.7654	0.5619	0.039*	
C17	0.81136 (17)	0.4608 (4)	0.53591 (10)	0.0266 (5)	
H17A	0.8654	0.4364	0.5181	0.032*	
H17B	0.8001	0.3288	0.5554	0.032*	
C18	0.49744 (17)	0.7909 (4)	0.39729 (10)	0.0227 (5)	
C19	0.41892 (18)	0.8393 (4)	0.34241 (11)	0.0322 (6)	
Cl1	0.43173 (7)	0.68095 (18)	0.28514 (4)	0.0736 (3)	
F7	0.33144 (11)	0.8114 (3)	0.35113 (8)	0.0503 (5)	
F8	0.42202 (13)	1.0418 (3)	0.32666 (8)	0.0548 (5)	
O2	0.47705 (12)	0.6592 (3)	0.43060 (7)	0.0345 (4)	
O3	0.57421 (12)	0.8831 (3)	0.40098 (7)	0.0337 (4)	

### Atomic displacement parameters (Å^2^)

**Table:**

	*U*^11^	*U*^22^	*U*^33^	*U*^12^	*U*^13^	*U*^23^
F1	0.0265 (8)	0.0331 (9)	0.0508 (10)	−0.0024 (7)	−0.0085 (7)	0.0049 (7)
F2	0.0410 (9)	0.0275 (8)	0.0417 (9)	0.0037 (7)	0.0150 (7)	0.0167 (7)
F3	0.0443 (9)	0.0331 (8)	0.0319 (8)	0.0130 (7)	0.0087 (7)	−0.0002 (7)
F4	0.0263 (8)	0.0453 (9)	0.0343 (8)	0.0014 (7)	0.0098 (6)	0.0052 (7)
F5	0.0402 (9)	0.0323 (8)	0.0296 (8)	0.0005 (7)	0.0111 (7)	−0.0070 (6)
F6	0.0478 (9)	0.0450 (9)	0.0241 (7)	0.0091 (8)	0.0172 (7)	0.0081 (7)
O1	0.0352 (10)	0.0257 (9)	0.0256 (9)	−0.0125 (8)	0.0129 (8)	−0.0029 (7)
N1	0.0215 (10)	0.0203 (10)	0.0209 (10)	−0.0036 (8)	0.0011 (8)	−0.0009 (8)
N2	0.0257 (11)	0.0225 (11)	0.0166 (9)	−0.0019 (9)	0.0039 (8)	0.0009 (8)
C1	0.0228 (11)	0.0176 (11)	0.0202 (11)	−0.0037 (10)	0.0016 (9)	0.0002 (9)
C2	0.0258 (12)	0.0234 (12)	0.0186 (11)	−0.0041 (10)	0.0040 (9)	0.0027 (10)
C3	0.0219 (12)	0.0217 (12)	0.0180 (11)	−0.0059 (10)	0.0035 (9)	−0.0026 (9)
C4	0.0209 (11)	0.0235 (12)	0.0171 (10)	−0.0023 (10)	0.0021 (9)	−0.0006 (9)
C5	0.0256 (12)	0.0230 (12)	0.0210 (11)	0.0011 (10)	0.0044 (9)	−0.0027 (10)
C6	0.0320 (13)	0.0250 (13)	0.0203 (11)	0.0041 (11)	0.0026 (10)	0.0009 (10)
C7	0.0347 (14)	0.0290 (13)	0.0169 (11)	0.0007 (11)	0.0047 (10)	0.0043 (10)
C8	0.0232 (12)	0.0243 (12)	0.0201 (11)	−0.0033 (10)	0.0037 (9)	−0.0027 (10)
C9	0.0181 (11)	0.0206 (12)	0.0185 (10)	−0.0038 (9)	0.0007 (9)	0.0003 (9)
C10	0.0239 (12)	0.0245 (12)	0.0257 (12)	−0.0036 (10)	0.0024 (10)	0.0023 (10)
C11	0.0304 (14)	0.0294 (13)	0.0236 (12)	0.0004 (11)	0.0080 (10)	0.0044 (11)
C12	0.0246 (12)	0.0229 (12)	0.0202 (11)	−0.0040 (10)	0.0072 (9)	0.0000 (9)
C13	0.0222 (11)	0.0227 (12)	0.0187 (11)	−0.0025 (10)	0.0062 (9)	0.0001 (9)
C14	0.0440 (15)	0.0227 (13)	0.0233 (12)	−0.0020 (11)	0.0108 (11)	−0.0034 (10)
C15	0.0474 (16)	0.0285 (13)	0.0187 (12)	−0.0084 (12)	0.0036 (11)	−0.0049 (10)
C16	0.0377 (14)	0.0314 (14)	0.0229 (12)	−0.0103 (12)	−0.0011 (11)	0.0012 (11)
C17	0.0281 (13)	0.0261 (13)	0.0228 (12)	−0.0036 (11)	0.0012 (10)	0.0020 (10)
C18	0.0257 (12)	0.0232 (12)	0.0210 (11)	0.0006 (10)	0.0089 (10)	−0.0049 (10)
C19	0.0324 (14)	0.0314 (15)	0.0304 (13)	0.0004 (12)	0.0034 (11)	0.0044 (11)
Cl1	0.0796 (6)	0.0932 (8)	0.0373 (5)	0.0119 (6)	−0.0064 (4)	−0.0325 (5)
F7	0.0260 (8)	0.0627 (12)	0.0579 (11)	0.0023 (8)	0.0018 (7)	0.0159 (9)
F8	0.0594 (11)	0.0444 (11)	0.0564 (11)	0.0056 (9)	0.0060 (9)	0.0235 (9)
O2	0.0284 (9)	0.0451 (11)	0.0310 (10)	0.0012 (9)	0.0093 (8)	0.0134 (9)
O3	0.0320 (10)	0.0380 (10)	0.0316 (10)	−0.0117 (9)	0.0085 (8)	−0.0078 (8)

### Geometric parameters (Å, º)

**Table:**

F1—C10	1.342 (3)	C7—C8	1.367 (3)
F2—C10	1.344 (3)	C7—H7	0.9500
F3—C10	1.329 (3)	C8—C9	1.428 (3)
F4—C11	1.331 (3)	C8—C11	1.502 (3)
F5—C11	1.346 (3)	C12—C13	1.536 (3)
F6—C11	1.350 (3)	C12—H12	1.0000
O1—C12	1.422 (3)	C13—C17	1.517 (3)
O1—H1O	0.835 (10)	C13—H13	1.0000
N1—C1	1.313 (3)	C14—C15	1.514 (4)
N1—C9	1.365 (3)	C14—H14A	0.9900
N2—C14	1.496 (3)	C14—H14B	0.9900
N2—C13	1.498 (3)	C15—C16	1.522 (4)
N2—H1N	0.888 (10)	C15—H15A	0.9900
N2—H2N	0.886 (10)	C15—H15B	0.9900
C1—C2	1.403 (3)	C16—C17	1.527 (3)
C1—C10	1.509 (3)	C16—H16A	0.9900
C2—C3	1.374 (3)	C16—H16B	0.9900
C2—H2	0.9500	C17—H17A	0.9900
C3—C4	1.423 (3)	C17—H17B	0.9900
C3—C12	1.517 (3)	C18—O2	1.238 (3)
C4—C5	1.418 (3)	C18—O3	1.238 (3)
C4—C9	1.429 (3)	C18—C19	1.540 (3)
C5—C6	1.368 (3)	C19—F8	1.341 (3)
C5—H5	0.9500	C19—F7	1.343 (3)
C6—C7	1.407 (3)	C19—Cl1	1.744 (3)
C6—H6	0.9500		
			
C12—O1—H1O	107 (2)	F6—C11—C8	111.3 (2)
C1—N1—C9	116.79 (19)	O1—C12—C3	112.03 (19)
C14—N2—C13	114.28 (19)	O1—C12—C13	105.27 (17)
C14—N2—H1N	108.3 (17)	C3—C12—C13	112.92 (18)
C13—N2—H1N	110.7 (17)	O1—C12—H12	108.8
C14—N2—H2N	108.9 (17)	C3—C12—H12	108.8
C13—N2—H2N	107.6 (16)	C13—C12—H12	108.8
H1N—N2—H2N	107 (2)	N2—C13—C17	109.37 (18)
N1—C1—C2	125.3 (2)	N2—C13—C12	106.47 (17)
N1—C1—C10	114.6 (2)	C17—C13—C12	115.3 (2)
C2—C1—C10	120.1 (2)	N2—C13—H13	108.5
C3—C2—C1	118.9 (2)	C17—C13—H13	108.5
C3—C2—H2	120.5	C12—C13—H13	108.5
C1—C2—H2	120.5	N2—C14—C15	110.1 (2)
C2—C3—C4	118.5 (2)	N2—C14—H14A	109.6
C2—C3—C12	120.2 (2)	C15—C14—H14A	109.6
C4—C3—C12	121.3 (2)	N2—C14—H14B	109.6
C5—C4—C3	123.7 (2)	C15—C14—H14B	109.6
C5—C4—C9	118.8 (2)	H14A—C14—H14B	108.1
C3—C4—C9	117.5 (2)	C14—C15—C16	111.5 (2)
C6—C5—C4	120.4 (2)	C14—C15—H15A	109.3
C6—C5—H5	119.8	C16—C15—H15A	109.3
C4—C5—H5	119.8	C14—C15—H15B	109.3
C5—C6—C7	120.8 (2)	C16—C15—H15B	109.3
C5—C6—H6	119.6	H15A—C15—H15B	108.0
C7—C6—H6	119.6	C15—C16—C17	110.9 (2)
C8—C7—C6	120.7 (2)	C15—C16—H16A	109.5
C8—C7—H7	119.6	C17—C16—H16A	109.5
C6—C7—H7	119.6	C15—C16—H16B	109.5
C7—C8—C9	120.0 (2)	C17—C16—H16B	109.5
C7—C8—C11	120.8 (2)	H16A—C16—H16B	108.1
C9—C8—C11	119.2 (2)	C13—C17—C16	111.3 (2)
N1—C9—C4	122.8 (2)	C13—C17—H17A	109.4
N1—C9—C8	118.1 (2)	C16—C17—H17A	109.4
C4—C9—C8	119.0 (2)	C13—C17—H17B	109.4
F3—C10—F1	106.86 (19)	C16—C17—H17B	109.4
F3—C10—F2	106.77 (19)	H17A—C17—H17B	108.0
F1—C10—F2	105.88 (19)	O2—C18—O3	128.6 (2)
F3—C10—C1	113.66 (19)	O2—C18—C19	116.1 (2)
F1—C10—C1	111.04 (19)	O3—C18—C19	115.3 (2)
F2—C10—C1	112.17 (19)	F8—C19—F7	105.5 (2)
F4—C11—F5	107.2 (2)	F8—C19—C18	111.2 (2)
F4—C11—F6	106.65 (19)	F7—C19—C18	111.5 (2)
F5—C11—F6	105.81 (19)	F8—C19—Cl1	108.24 (18)
F4—C11—C8	113.7 (2)	F7—C19—Cl1	109.36 (19)
F5—C11—C8	111.7 (2)	C18—C19—Cl1	110.85 (18)
			
C9—N1—C1—C2	2.6 (3)	N1—C1—C10—F2	−158.96 (19)
C9—N1—C1—C10	−174.73 (19)	C2—C1—C10—F2	23.6 (3)
N1—C1—C2—C3	−2.4 (3)	C7—C8—C11—F4	117.4 (2)
C10—C1—C2—C3	174.7 (2)	C9—C8—C11—F4	−64.5 (3)
C1—C2—C3—C4	−0.9 (3)	C7—C8—C11—F5	−121.2 (2)
C1—C2—C3—C12	177.0 (2)	C9—C8—C11—F5	57.0 (3)
C2—C3—C4—C5	−175.6 (2)	C7—C8—C11—F6	−3.1 (3)
C12—C3—C4—C5	6.5 (3)	C9—C8—C11—F6	175.05 (19)
C2—C3—C4—C9	3.7 (3)	C2—C3—C12—O1	−20.3 (3)
C12—C3—C4—C9	−174.2 (2)	C4—C3—C12—O1	157.6 (2)
C3—C4—C5—C6	179.0 (2)	C2—C3—C12—C13	98.3 (2)
C9—C4—C5—C6	−0.3 (3)	C4—C3—C12—C13	−83.8 (3)
C4—C5—C6—C7	2.4 (4)	C14—N2—C13—C17	56.2 (3)
C5—C6—C7—C8	−0.6 (4)	C14—N2—C13—C12	−178.61 (18)
C6—C7—C8—C9	−3.3 (4)	O1—C12—C13—N2	−59.7 (2)
C6—C7—C8—C11	174.9 (2)	C3—C12—C13—N2	177.79 (18)
C1—N1—C9—C4	0.6 (3)	O1—C12—C13—C17	61.8 (2)
C1—N1—C9—C8	179.8 (2)	C3—C12—C13—C17	−60.7 (3)
C5—C4—C9—N1	175.6 (2)	C13—N2—C14—C15	−55.7 (3)
C3—C4—C9—N1	−3.7 (3)	N2—C14—C15—C16	54.0 (3)
C5—C4—C9—C8	−3.5 (3)	C14—C15—C16—C17	−55.1 (3)
C3—C4—C9—C8	177.2 (2)	N2—C13—C17—C16	−55.3 (3)
C7—C8—C9—N1	−173.9 (2)	C12—C13—C17—C16	−175.15 (19)
C11—C8—C9—N1	7.9 (3)	C15—C16—C17—C13	56.0 (3)
C7—C8—C9—C4	5.3 (3)	O2—C18—C19—F8	146.3 (2)
C11—C8—C9—C4	−172.9 (2)	O3—C18—C19—F8	−36.6 (3)
N1—C1—C10—F3	−37.7 (3)	O2—C18—C19—F7	28.8 (3)
C2—C1—C10—F3	144.8 (2)	O3—C18—C19—F7	−154.0 (2)
N1—C1—C10—F1	82.8 (2)	O2—C18—C19—Cl1	−93.3 (2)
C2—C1—C10—F1	−94.7 (3)	O3—C18—C19—Cl1	83.9 (2)

### Hydrogen-bond geometry (Å, º)

Cg1 is the centroid of the (C4–C9) ring.

**Table:**

*D*—H···*A*	*D*—H	H···*A*	*D*···*A*	*D*—H···*A*
N2—H2*N*···O1	0.89 (2)	2.34 (2)	2.722 (3)	106 (2)
O1—H1*O*···O3^i^	0.84 (2)	1.83 (2)	2.668 (3)	178 (3)
N2—H1*N*···O2	0.89 (2)	1.92 (2)	2.808 (3)	177 (2)
N2—H2*N*···O2^ii^	0.89 (2)	2.05 (2)	2.776 (3)	138 (2)
C5—H5···O3	0.95	2.45	3.367 (3)	162
C14—H14*B*···O1^iii^	0.99	2.39	3.362 (3)	166
C19—Cl1···*Cg*1^iv^	1.74 (1)	3.91 (1)	4.208 (3)	88 (1)
C10—F3···*Cg*1^i^	1.33 (1)	3.09 (1)	3.762 (3)	110 (1)

Symmetry codes: (i) *x*, *y*−1, *z*; (ii) −*x*+1, −*y*+1, −*z*+1; (iii) *x*, *y*+1, *z*; (iv) −*x*+1, *y*+1/2, −*z*+1/2.
